# Impacts of Anthropogenic Activities and Climate Change on Forage Nutrition Storage in Tibetan Grasslands

**DOI:** 10.3390/plants12142735

**Published:** 2023-07-23

**Authors:** Shaowei Li, Gang Fu

**Affiliations:** Lhasa Plateau Ecosystem Research Station, Key Laboratory of Ecosystem Network Observation and Modeling, Institute of Geographic Sciences and Natural Resources Research, Chinese Academy of Sciences, Beijing 100101, China; leesw@igsnrr.ac.cn

**Keywords:** asymmetrical influence, asymmetrical response, warming, cooling, precipitation change, dimming

## Abstract

Uncertainties about the impacts of anthropogenic activities and climate change on forage nutrition storage of grasslands can limit the adaptive management of grasslands across the whole Tibetan Plateau. The main objective was to investigate the impacts of anthropogenic activities and climate change on the forage nutrition storage of grasslands on the Tibetan Plateau. Based on random forest models, we quantified the responses of forage nutrition storage to anthropogenic activities and climate change across the whole Tibetan grasslands from 2000 to 2020. Warming and increased precipitation did not always increase forage nutrition storage, and cooling and decreased precipitation did not always reduce forage nutrition storage. Compared to temperature and precipitation changes, radiation change had stronger contributions to potential and actual forage nutrition storage. Humankind’s activities altered the impacts of climate change on forage nutrition storage. The impacts of anthropogenic activities on forage nutrition storage increased linearly with increasing mean annual temperature and decreasing elevation but showed quadratic relationships with longitude, mean annual precipitation and radiation. The change in the impacts of humankind’s activities on forage nutrition storage was more closely related to radiation change than temperature and precipitation changes. The findings observed by this study caution that the impacts of radiation change on forage nutrition forage should be taken seriously under global change. Both climate change and humankind activities cannot always increase forage nutrition storage but may cause the degradation of forage nutrition storage.

## 1. Introduction

The ecological livestock carrying capacity of grasslands refers to the ability to sustainably carry domestic animal aggregates, grazing intensity and conservation of wildlife under the conditions of ensuring the rational exploitation and utilization of forage resources, and the good and circular development of the ecological environment for a particular region of grassland ecosystem in a certain period. Rational ecological livestock carrying capacity is the guarantee to realize a forage-livestock balance in a variety of grassland ecosystems at diverse temporal and spatial scales. Accurate quantification of forage storage is an important aspect to achieve targeted rational ecological livestock carrying capacity. Understanding the influences of anthropogenic activities and climate change on forage yield and related causes can help predict the changes in the ecological livestock carrying capacity and, in turn, the impacts on human living standards and social high-quality development under global change scenes. From this perspective, research has been or is being conducted on the influences of anthropogenic activities and/or climate change on forage storage and livestock carrying capacity [1,2,3,4]. However, a few uncertainties are still present. Firstly, forage yield and nutrition quality are two important characteristics associated with forage storage [5,6,7], whereas forage nutrition storage is the key comprehensive variable of forage yield and nutrition quality [1,4,8,9]. Compared to forage yield and nutrition quality, a few studies have studied the response of forage nutrition storage to anthropogenic activities and climate change, especially for the relative long-term temporal scale and/or large spatial scale [8,10,11]. Secondly, it is probable that the current forage-livestock balance policies are primarily based on herbage hay storage (i.e., hay-carrying capacity) rather than forage nutrition storage (i.e., nutrition-carrying capacity). The hay carrying capacity is not always equal to the nutrition carrying capacity [11,12]. When hay carrying capacity is greater than nutrition-carrying capacity, the livestock are malnourished and less able to resist external stresses [13]. In contrast, when hay-carrying capacity is lower than nutrition carrying capacity, the carrying capacity of grasslands is probably highly underestimated and grasslands forage resources are probably highly wasted [14]. All these probably increase the uncertainty of the forage-livestock balance, which may be dampened by understanding the influences of anthropogenic activities and climate change on forage nutrition storage and associated driving causes. Hence, extra research on the response of forage nutrition storage to anthropogenic activities and climate change is needed.

The Tibetan Plateau is one of the main living areas of the Tibetan compatriots in China, and with the overall poverty alleviation, the living standard of the Tibetan compatriots has again reached a new level. However, agriculture and husbandry are still vital sources of income for Tibetan farmers and herdsmen in the farming-pastoral areas, and husbandry is an important source of income in the pastoral areas. Grassland ecosystems and their derived various resources (e.g., forage yield), which are obviously influenced by anthropogenic activities and climate change, are still extremely valuable places and resources for the survival of the Tibetan compatriots. Accordingly, understanding the reactions of grassland ecosystems to global change and the associated driving causes is still extremely important for the high-quality development of grassland husbandry and further provisions of living standards for the Tibetan compatriots. On the other hand, with the implementation of various ecological protection measures and warming and wetting climate change, grassland ecosystems of the Tibetan Plateau have undergone remarkable changes with an overall increase in forage yield but overall reductions in forage crude protein (CP) and crude ash (Ash) content [15,16]. The increase in forage yield of grassland ecosystems seems to imply that the Tibetan compatriots can probably raise more livestock and earn more income. In contrast, the reductions in forage CP and Ash content of grassland ecosystems seem to imply that the livestock is most likely to be malnourished, even though there may be enough fed. This event likely causes a decline in the quality of livestock products and, in turn, the income of farmers and herdsmen. Meanwhile, no research has investigated the impacts of anthropogenic activities and climate change on forage nutrition storage in grassland ecosystems on the whole Tibetan Plateau scale, which in turn probably greatly increase the uncertainty of the net influences of the anthropogenic activities and climate change on the livestock and in turn the total income of the Tibetan compatriots over the whole grassland areas of the Tibetan Plateau. Therefore, extra research is highly needed to better serve the high-quality development of husbandry and income of farmers and herdsmen on the Tibetan Plateau.

Here, it was explored that the influences of anthropogenic activities and climate change on forage nutrition storage of the grasslands on the whole Tibetan Plateau scale during 2000–2020. We made four hypotheses (i.e., *H1*, *H2*, *H3* and *H4*). Firstly, although the climate conditions on the Tibetan Plateau cause warming, wetting and dimming, on the whole, it is cooling, drying and brightening in some areas [15]. The opposite climate change trends (i.e., warming versus cooling, increased precipitation and decreased precipitation, dimming and brightening) can have asymmetric influences on plant growth and nutrition quality on the Tibetan Plateau [15,17,18,19]. Thus, we hypothesized that warming/wetting/dimming and cooling/drying/brightening had asymmetrical influences on forage nutrition storage (*H1*). Secondly, both low temperature and drought can limit plant growth [20,21], thus we hypothesized that warming or increased precipitation can increase forage nutrition storage, but cooling or decreased precipitation can reduce forage nutrition storage (*H2*). Thirdly, temperature sensitivities of grassland ecosystems are generally negatively correlated with warming magnitudes [22,23], thus we hypothesized that the response of forage nutrition storage declined with increasing warming magnitude (*H3*). Fourthly, we hypothesized that the responses of forage nutritional pools to human activities can vary with geographical location, climatic background and climate change (*H4*).

## 2. Results

### 2.1. Variation Rates for Forage Nutrition Storage, and Their Correlations with Environmental Variables

**Figure A1:**
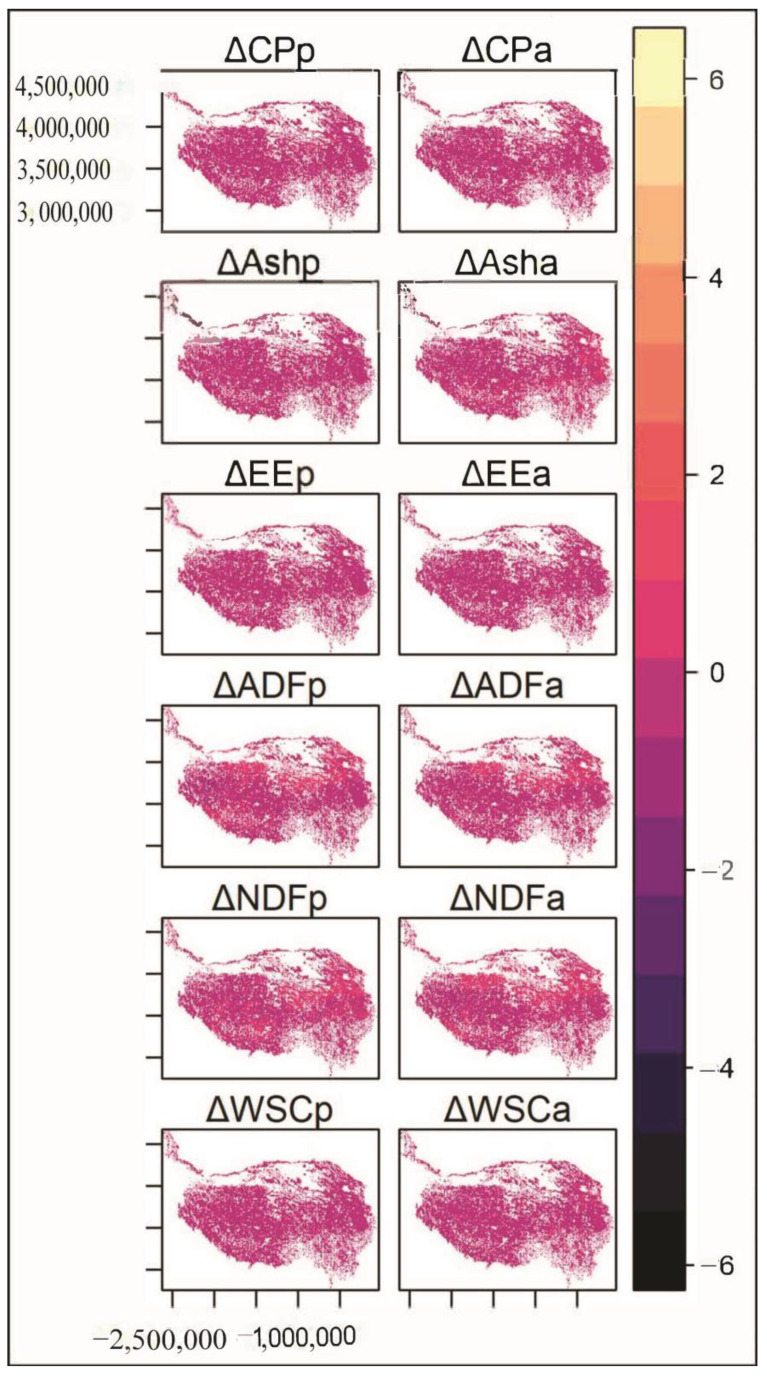
Spatial trend for the variation amplitude of potential crude protein storage (ΔCP_p_), actual crude protein storage (ΔCP_a_), potential crude ash storage (ΔAsh_p_), actual crude ash storage (ΔAsh_a_), potential ether extract storage (ΔEE_p_), actual ether extract storage (ΔEE_a_), potential acid detergent fiber storage (ΔADF_p_), actual acid detergent fiber storage (ΔADF_a_), potential neutral detergent fiber storage (ΔNDF_p_), actual neutral detergent fiber storage (ΔNDF_a_), potential water-soluble carbohydrate storage (ΔWSCp) and actual water-soluble carbohydrate storage (ΔWSCa), respectively.

Overall, the spatially averages of CP_p_, EE_p_, ADF_p_, NDF_p_, CP_a_, Ash_a_, EE_a_, ADF_a_, NDF_a_ and WSC_a_ showed an increasing trend with a rate of 0.004 g m^−2^ yr^−1^, 0.002 g m^−2^ yr^−1^, 0.034 g m^−2^ yr^−1^, 0.059 g m^−2^ yr^−1^, 0.017 g m^−2^ yr^−1^, 0.050 g m^−2^ yr^−1^, 0.004 g m^−2^ yr^−1^, 0.046 g m^−2^ yr^−1^, 0.079 g m^−2^ yr^−1^ and 0.011 g m^−2^ yr^−1^, respectively. In contrast, the spatially averages of Ash_p_ and WSC_p_ showed a decreasing trend with a rate of –0.003 g m^−2^ yr^−1^ and –0.002 g m^−2^ yr^−1^, respectively. Actually, climate change did not always increase the CP_p_, EE_p_, ADF_p_ and NDF_p_, and did not always decrease Ash_p_ and WSC_p_ (Figure A1). More than one-third of the areas CP_p_, EE_p_, ADF_p_ and NDF_p_ showed decreasing trends, but more than 48% of the areas Ash_p_ and WSC_p_ showed increasing trends (Figure A1). On the other hand, climate change and humankind activities together did not always increase the CP_a_, Ash_a_, EE_a_, ADF_a_, NDF_a_ and WSC_a_ (Figure A1). More than 32% of the areas CP_a_, Ash_a_, EE_a_, ADF_a_, NDF_a_ and WSC_a_ showed decreasing trends (Figure A1).

**Figure A2:**
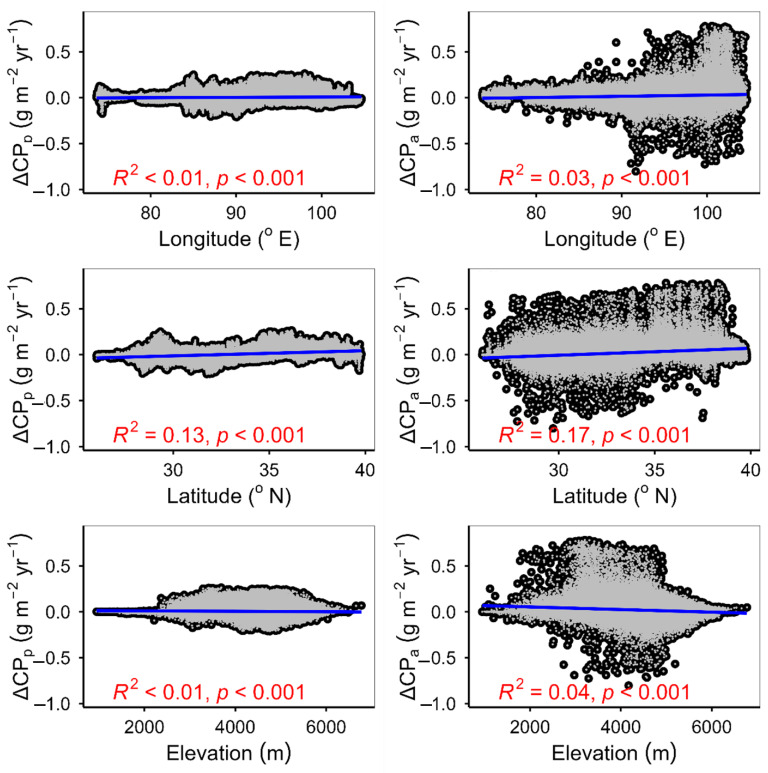
Correlations between the variation amplitudes of potential or actual crude protein storage (ΔCPp or ΔCPa) versus geography variables (i.e., longitude, latitude and elevation). The blue lines indicate the fitting lines.

**Figure A3:**
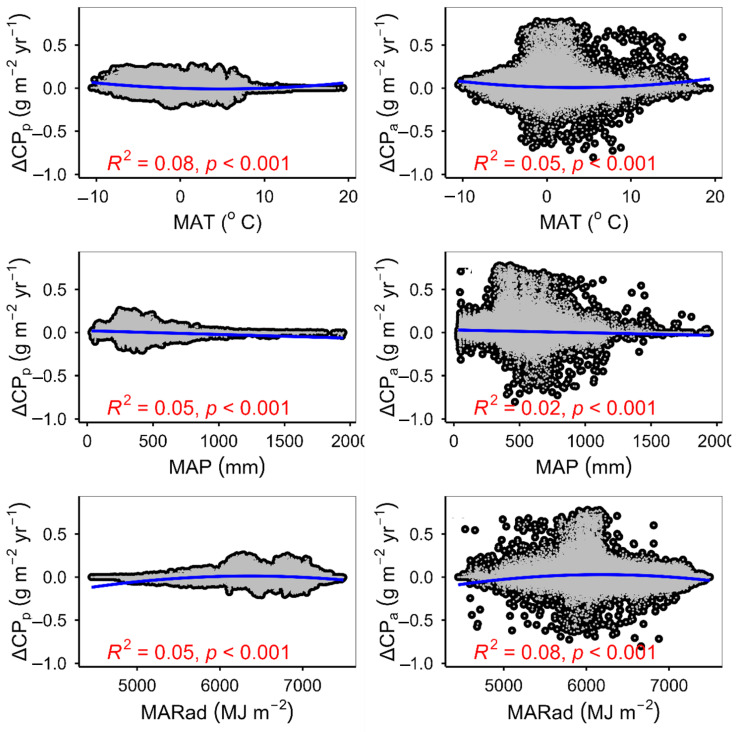
Correlations between the variation amplitudes of potential or actual crude protein storage (ΔCP_p_ or ΔCP_a_) versus climatic variables (i.e., MAP: mean annual precipitation, MAT: mean annual temperature, MARad: mean annual radiation). The blue lines indicate the fitting lines.

**Figure A4:**
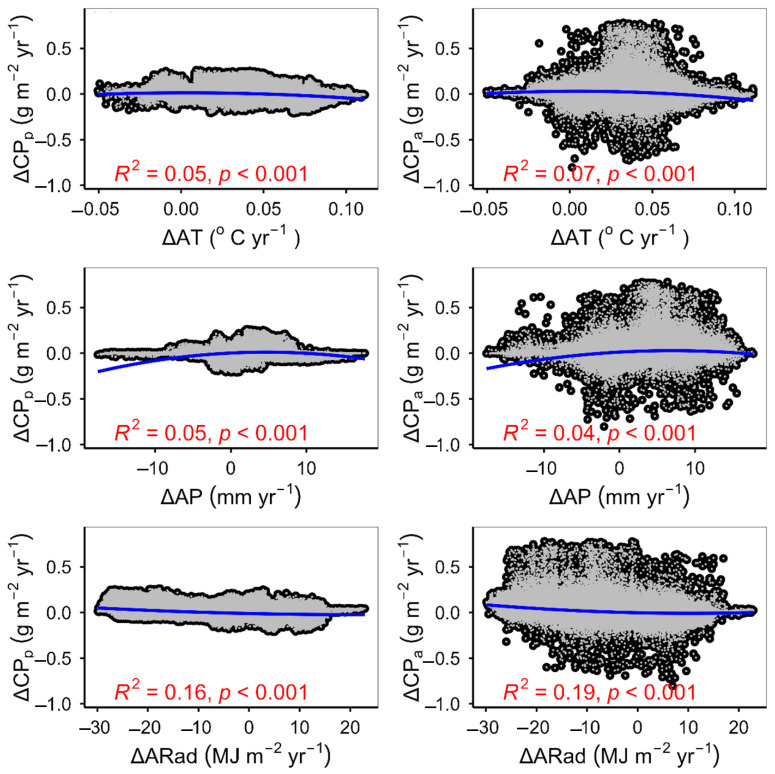
Correlations between the variation amplitudes of potential or actual crude protein storage (ΔCP_p_ or ΔCP_a_) versus the variation amplitudes of annual precipitation, annual temperature and annual radiation (i.e., ΔAT, ΔAP and ΔARad). The blue lines indicate the fitting lines.

**Figure A5:**
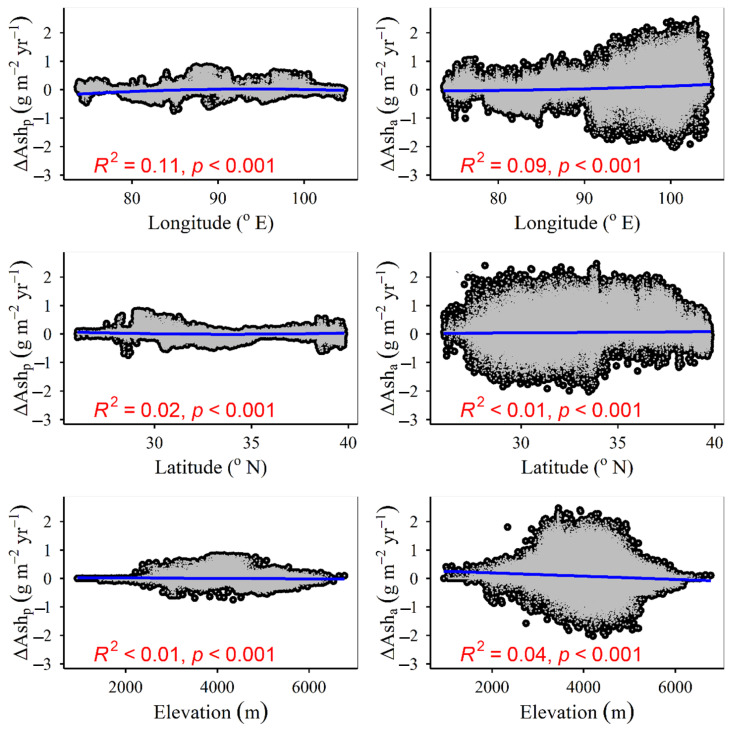
Correlations between the variation amplitudes of potential or actual crude ash storage (ΔAsh_p_ or ΔAsh_a_) versus geography variables (i.e., longitude, latitude and elevation). The blue lines indicate the fitting lines.

**Figure A6:**
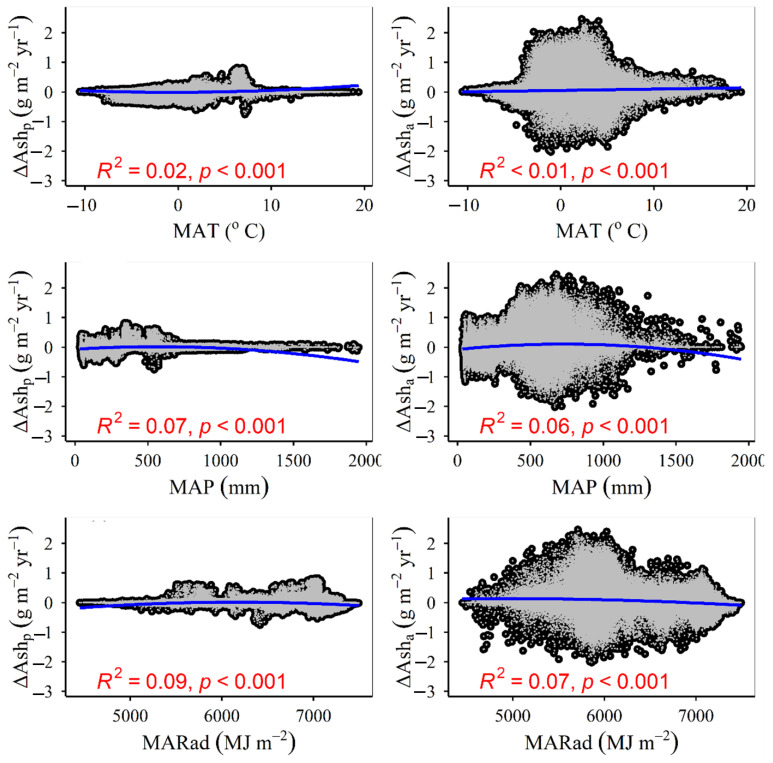
Correlations between the variation amplitudes of potential or actual crude ash storage (ΔAsh_p_ or ΔAsh_a_) versus climatic variables (i.e., MAP: mean annual precipitation, MAT: mean annual temperature, MARad: mean annual radiation). The blue lines indicate the fitting lines.

**Figure A7:**
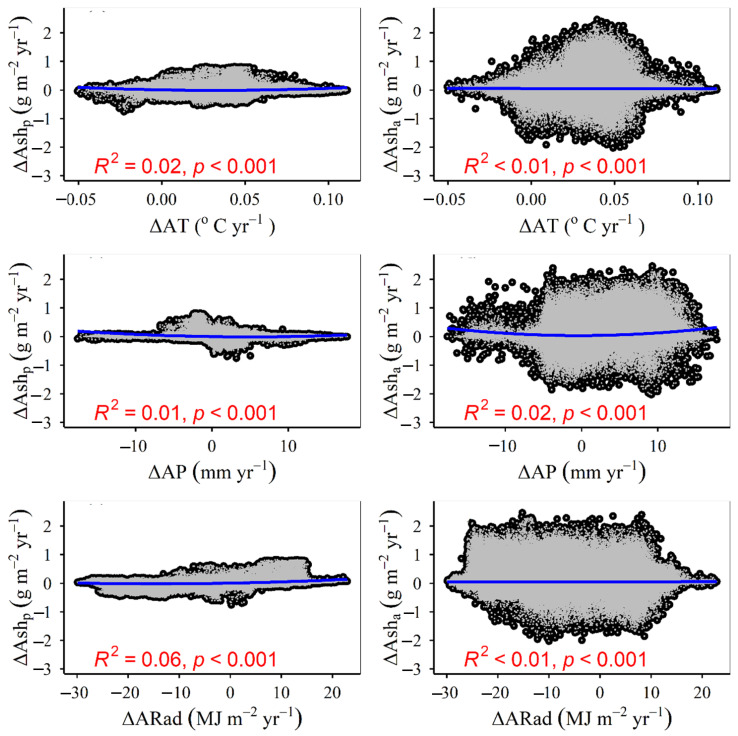
Correlations between the variation amplitudes of potential or actual crude ash storage (ΔAsh_p_ or ΔAsh_a_) versus the variation amplitudes of annual precipitation, annual temperature and annual radiation (i.e., ΔAT, ΔAP and ΔARad). The blue lines indicate the fitting lines.

**Figure A8:**
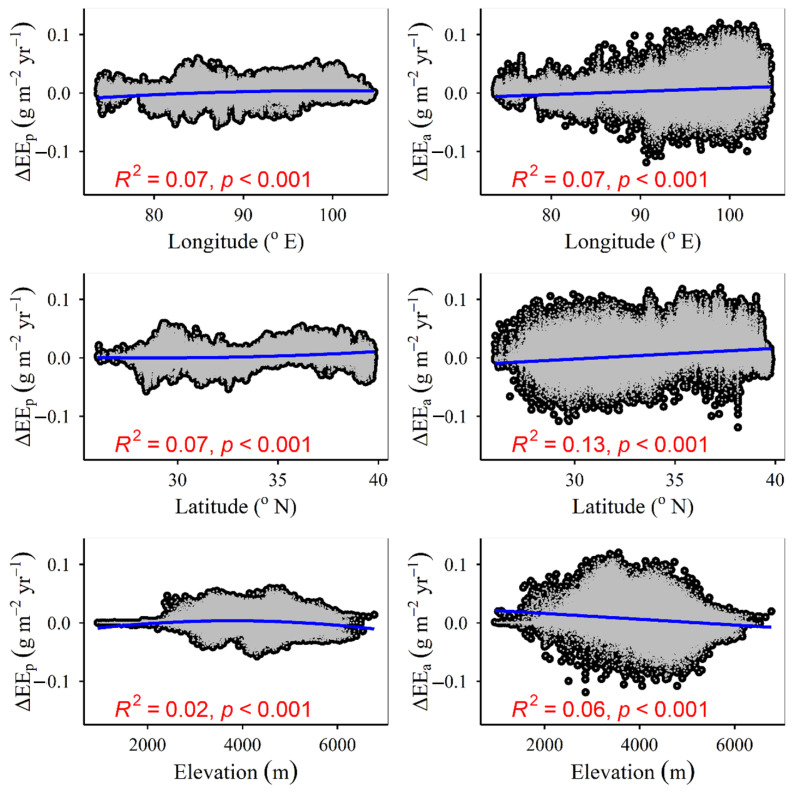
Correlations between the variation amplitudes of potential or actual ether extract storage (ΔEE_p_ or ΔEE_a_) versus geography variables (i.e., longitude, latitude and elevation). The blue lines indicate the fitting lines.

**Figure A9:**
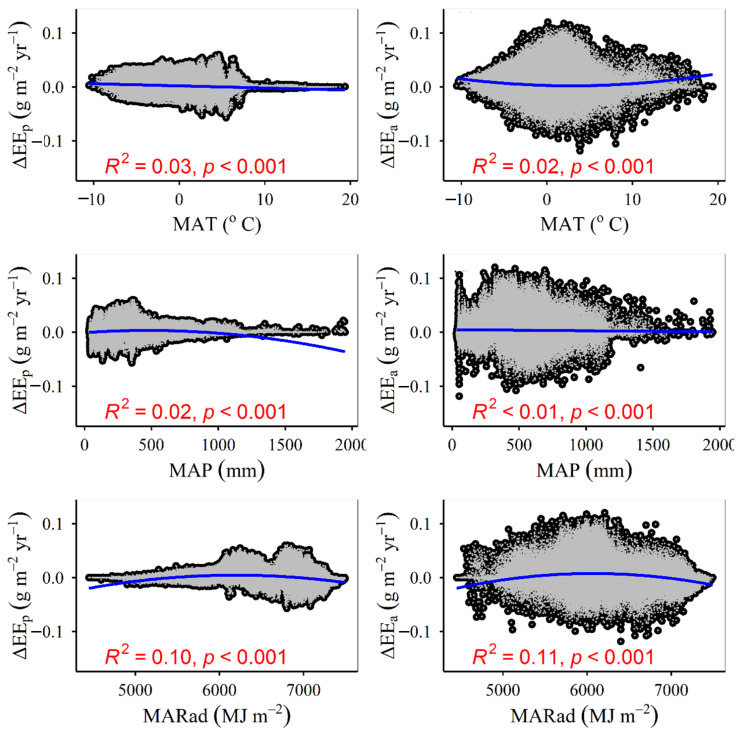
Correlations between the variation amplitudes of potential or actual ether extract storage (ΔEE_p_ or ΔEE_a_) versus climatic variables (i.e., MAP: mean annual precipitation, MAT: mean annual temperature, MARad: mean annual radiation). The blue lines indicate the fitting lines.

**Figure A10:**
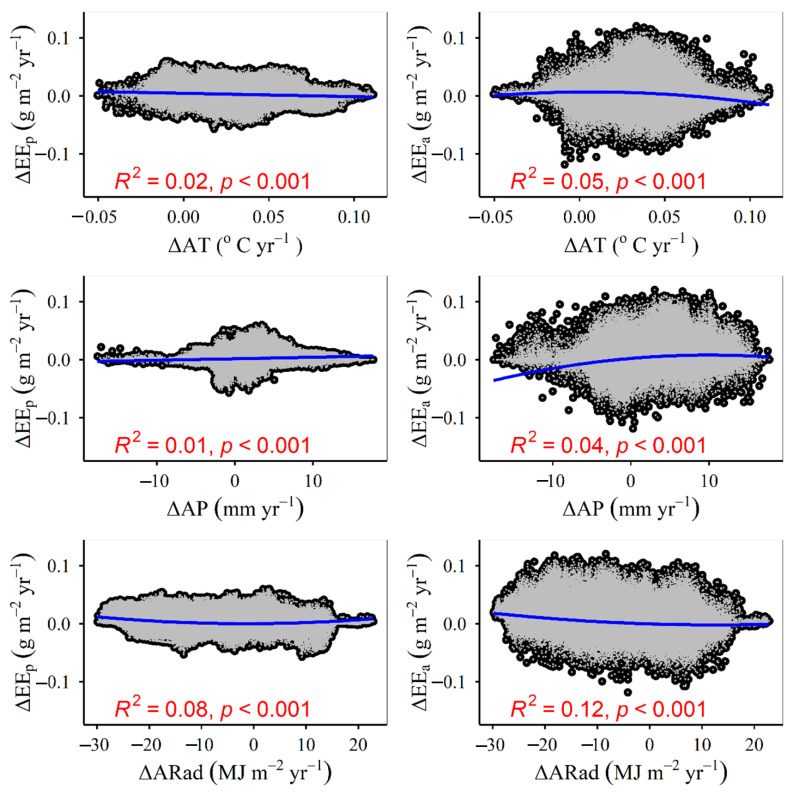
Correlations between the variation amplitudes of potential or actual ether extract storage (ΔEE_p_ or ΔEE_a_) versus the variation amplitudes of annual precipitation, annual temperature and annual radiation (i.e., ΔAT, ΔAP and ΔARad). The blue lines indicate the fitting lines.

**Figure A11:**
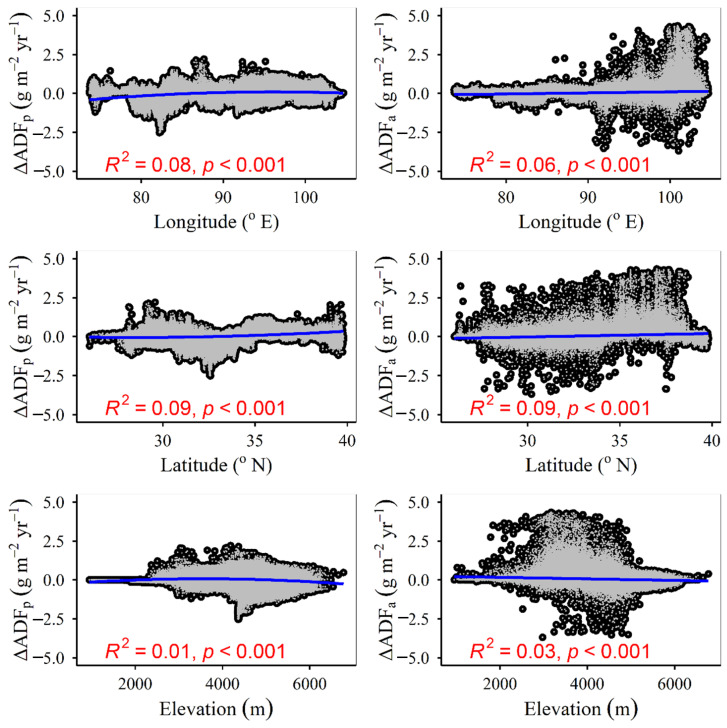
Correlations between the variation amplitudes of potential or actual acid detergent fiber storage (ΔADF_p_ or ΔADF_a_) versus geography variables (i.e., longitude, latitude and elevation). The blue lines indicate the fitting lines.

**Figure A12:**
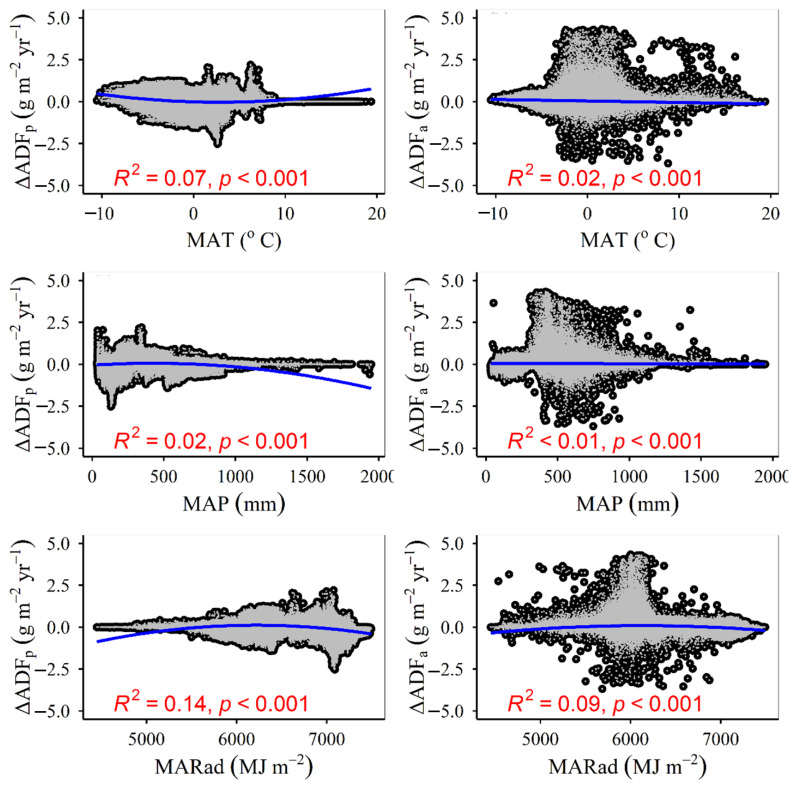
Correlations between the variation amplitudes of potential or actual acid detergent fiber storage (ΔADF_p_ or ΔADF_a_) versus climatic variables (i.e., MAP: mean annual precipitation, MAT: mean annual temperature, MARad: mean annual radiation). The blue lines indicate the fitting lines.

**Figure A13:**
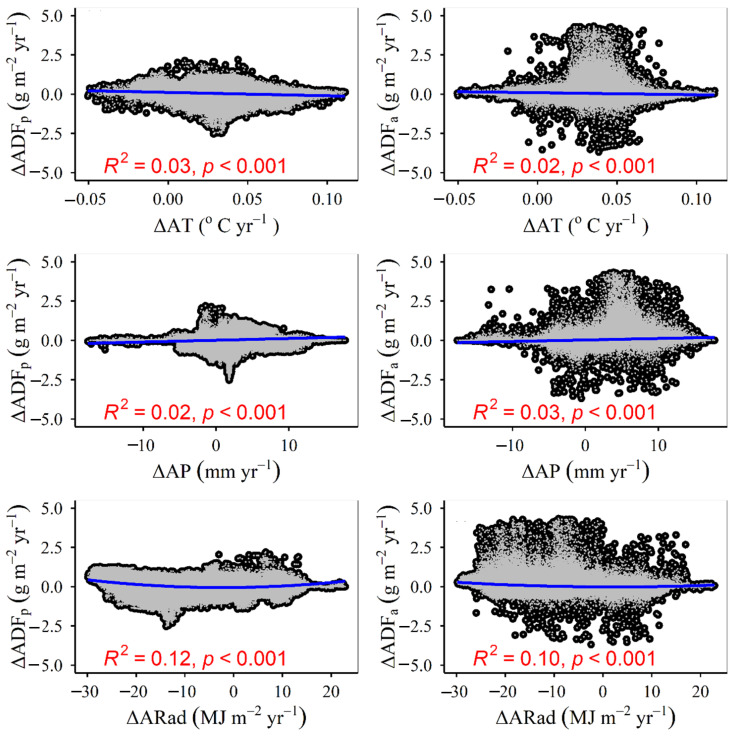
Correlations between the variation amplitudes of potential or actual acid detergent fiber storage (ΔADF_p_ or ΔADF_a_) versus the variation amplitudes of annual precipitation, annual temperature and annual radiation (i.e., ΔAT, ΔAP and ΔARad). The blue lines indicate the fitting lines.

**Figure A14:**
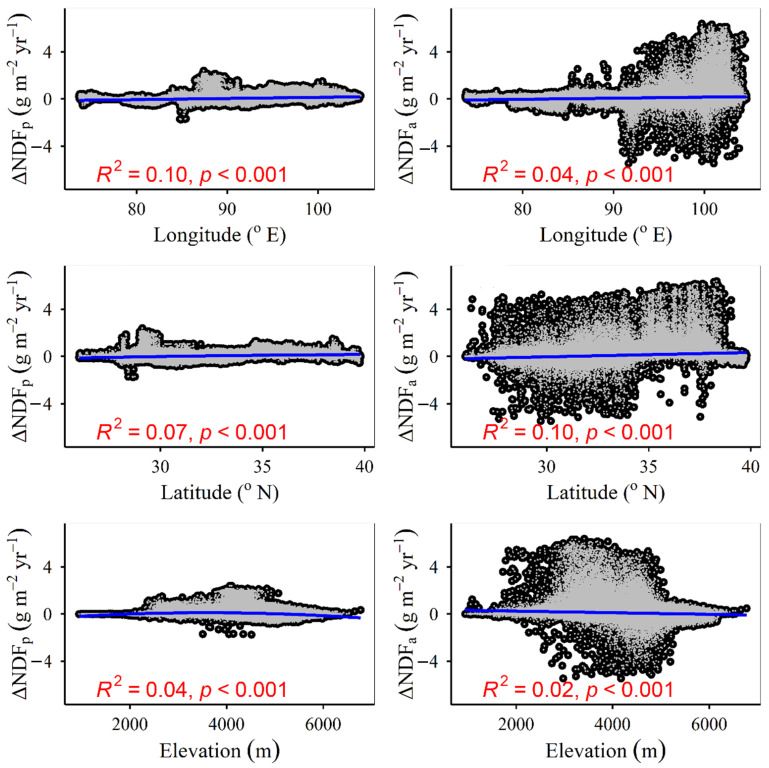
Correlations between the variation amplitudes of potential or actual neutral detergent fiber storage (ΔNDF_p_ or ΔNDF_a_) versus geography variables (i.e., longitude, latitude and elevation). The blue lines indicate the fitting lines.

**Figure A15:**
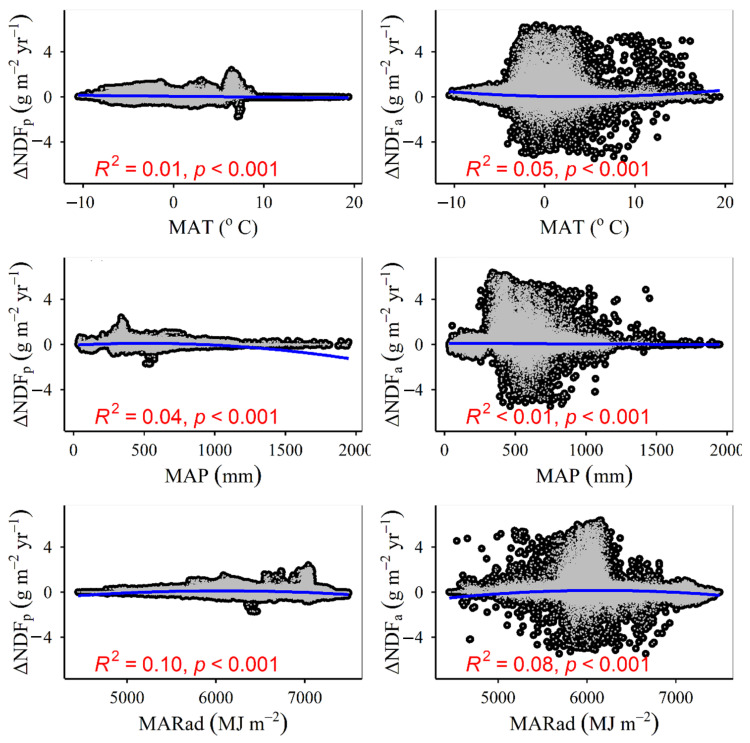
Correlations between the variation amplitudes of potential or actual neutral detergent fiber storage (ΔNDF_p_ or ΔNDF_a_) versus climatic variables (i.e., MAP: mean annual precipitation, MAT: mean annual temperature, MARad: mean annual radiation). The blue lines indicate the fitting lines.

**Figure A16:**
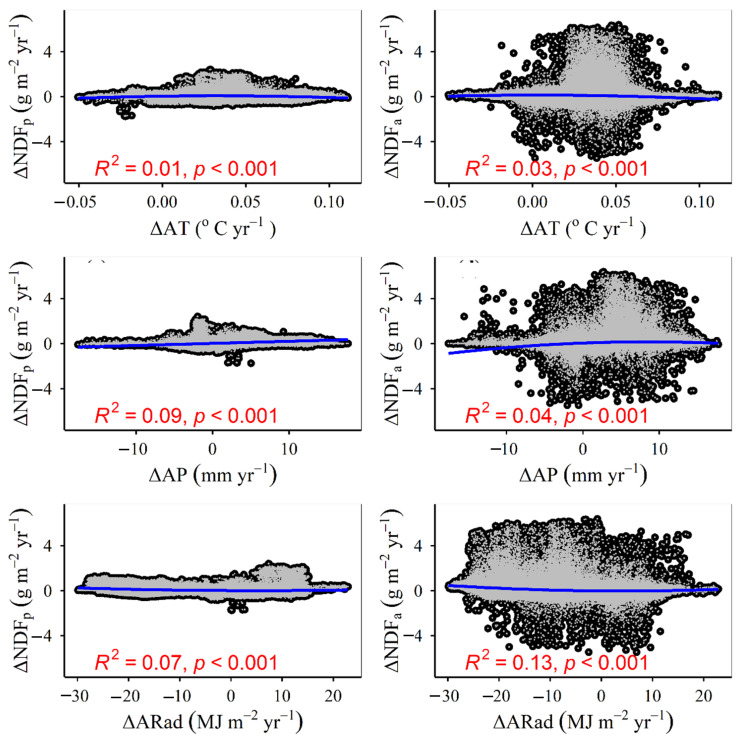
Correlations between the variation amplitudes of potential or actual neutral detergent fiber storage (ΔNDF_p_ or ΔNDF_a_) versus the variation amplitudes of annual precipitation, annual temperature and annual radiation (i.e., ΔAT, ΔAP and ΔARad). The blue lines indicate the fitting lines.

**Figure A17:**
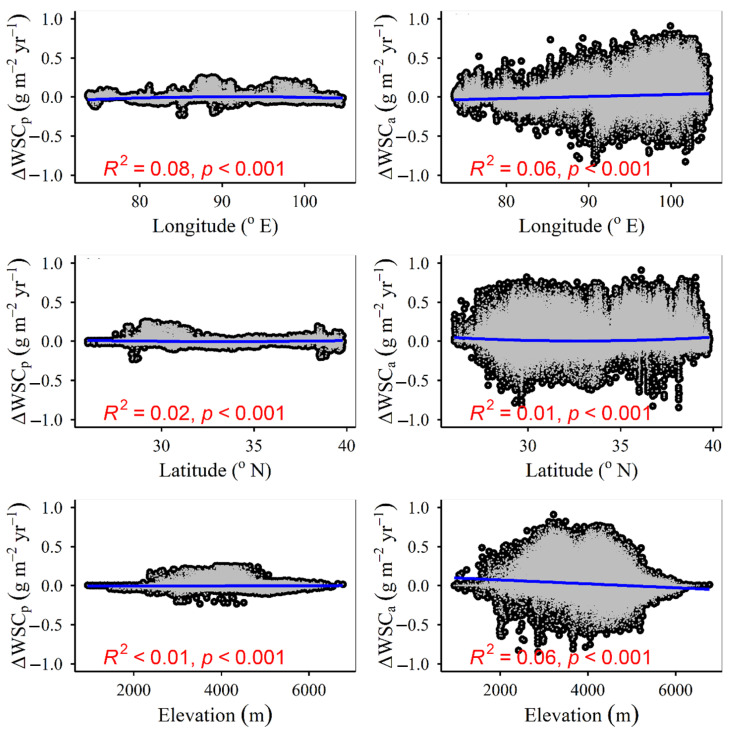
Correlations between the variation amplitudes of potential or actual water-soluble carbohydrate storage (ΔWSC_p_ or ΔWSC_a_) versus geography variables (i.e., longitude, latitude and elevation). The blue lines indicate the fitting lines.

**Figure A18:**
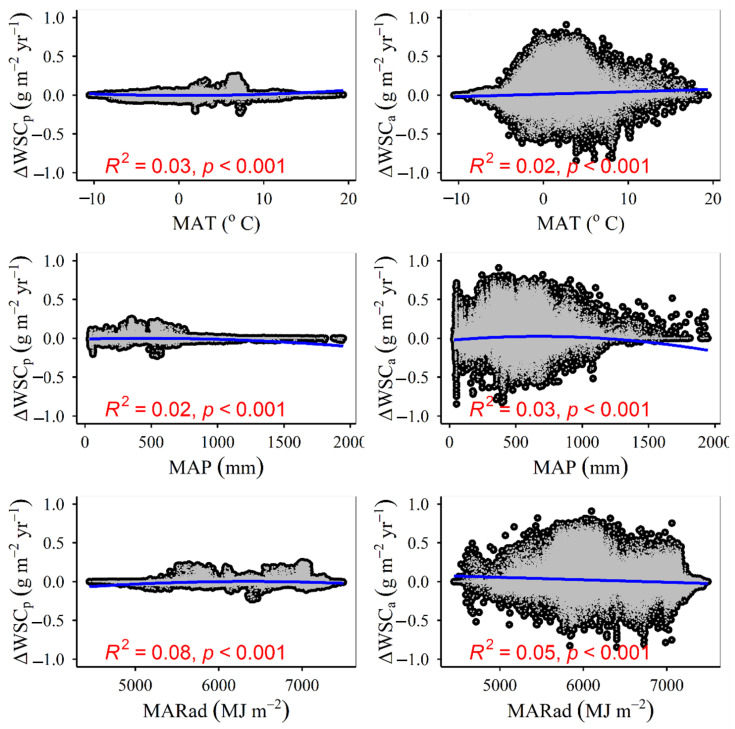
Correlations between the variation amplitudes of potential or actual water-soluble carbohydrate storage (ΔWSC_p_ or ΔWSC_a_) versus climatic variables (i.e., MAP: mean annual precipitation, MAT: mean annual temperature, MARad: mean annual radiation). The blue lines indicate the fitting lines.

**Figure A19:**
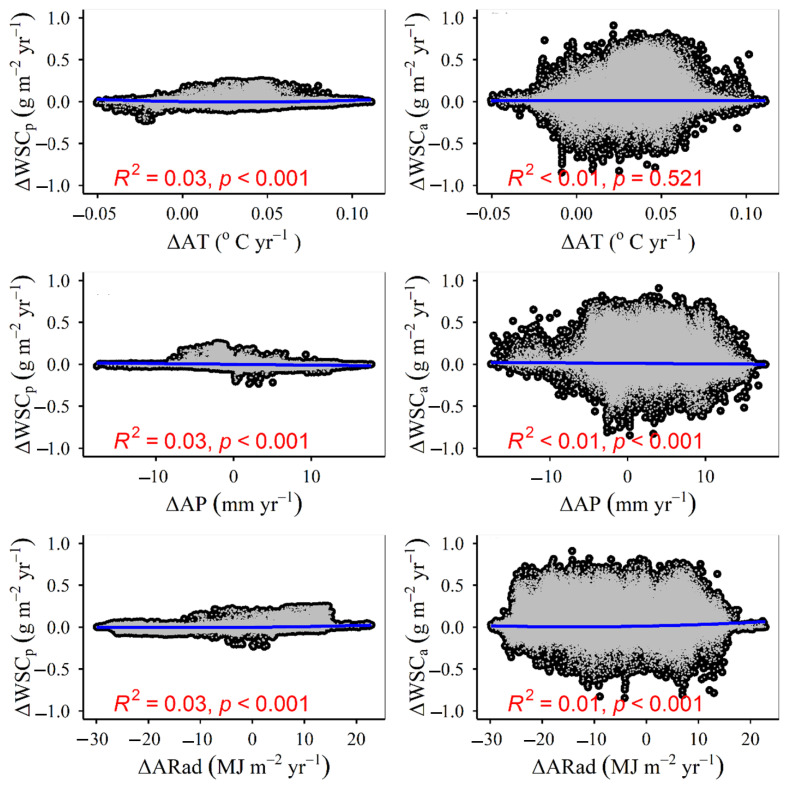
Correlations between the variation amplitudes of potential or actual water-soluble carbohydrate storage (ΔWSC_p_ or ΔWSC_a_) versus the variation amplitudes of annual precipitation, annual temperature and annual radiation (i.e., ΔAT, ΔAP and ΔARad). The blue lines indicate the fitting lines.

Although most of the ΔCP_p_, ΔAsh_p_, ΔEE_p_, ΔADF_p_, ΔNDF_p_, ΔWSC_p_, ΔCP_a_, ΔAsh_a_, ΔEE_a_, ΔADF_a_, ΔNDF_a_ and ΔWSC_a_ showed significant (*p* < 0.001) correlations with environmental variables, some of the *R*^2^ values were relatively low and even lower than 0.01 (Figure A2, Figure A3, Figure A4, Figure A5, Figure A6, Figure A7, Figure A8, Figure A9, Figure A10, Figure A11, Figure A12, Figure A13, Figure A14, Figure A15, Figure A16, Figure A17, Figure A18 and Figure A19), indicating a single environmental variable may have less explanatory power for forage nutritional pools. The *R*^2^ values of ΔCP_p_, ΔAsh_p_, ΔEE_p_, ΔADF_p_, ΔNDF_p_ and ΔWSC_p_ with environmental variables, were different from the *R*^2^ values of ΔCP_a_, ΔAsh_a_, ΔEE_a_, ΔADF_a_, ΔNDF_a_ and ΔWSC_a_ with environmental variables, respectively, (Figure A2, Figure A3, Figure A4, Figure A5, Figure A6, Figure A7, Figure A8, Figure A9, Figure A10, Figure A11, Figure A12, Figure A13, Figure A14, Figure A15, Figure A16, Figure A17, Figure A18 and Figure A19), indicating potential and actual forage nutritional pools differed in how closely they are related to environmental variables. Compared to latitude and elevation, longitude had closer correlations with ΔAsh_p_, ΔNDF_p_, ΔWSC_p_ and ΔAsh_a_ (Figure A5, Figure A14 and Figure A17). In contrast, compared to longitude and elevation, latitude had closer correlations with ΔCP_p_, ΔADF_p_, ΔCP_a_, ΔEE_a_, ΔADF_p_ and ΔNDF_a_ (Figure A2, Figure A8, Figure A11 and Figure A14). Compared to MAT and MAP, MARad had closer correlations with ΔCP_a_, ΔAsh_p_, ΔAsh_a_, ΔEE_p_, ΔEE_a_, ΔADF_p_, ΔADF_a_, ΔNDF_p_, ΔNDF_a_, ΔWSC_p_ and ΔWSC_a_ (Figure A3, Figure A6, Figure A9, Figure A12, Figure A15 and Figure A18). In contrast, compared to MAP and MARad, MAT had closer correlations with ΔCP_p_ (Figure A3). Compared to temperature and precipitation change, radiation change had closer correlations with ΔCP_p_, ΔCP_a_, ΔAsh_p_, ΔEE_p_, ΔEE_a_, ΔADF_p_, ΔADF_a_, ΔNDF_a_, and ΔWSC_a_ (Figure A4, Figure A7, Figure A10, Figure A13, Figure A16 and Figure A19). In contrast, compared to temperature and radiation change, precipitation change had closer correlations with ΔAsh_a_ and ΔNDF_p_ (Figure A7 and Figure A16).

**Figure 1:**
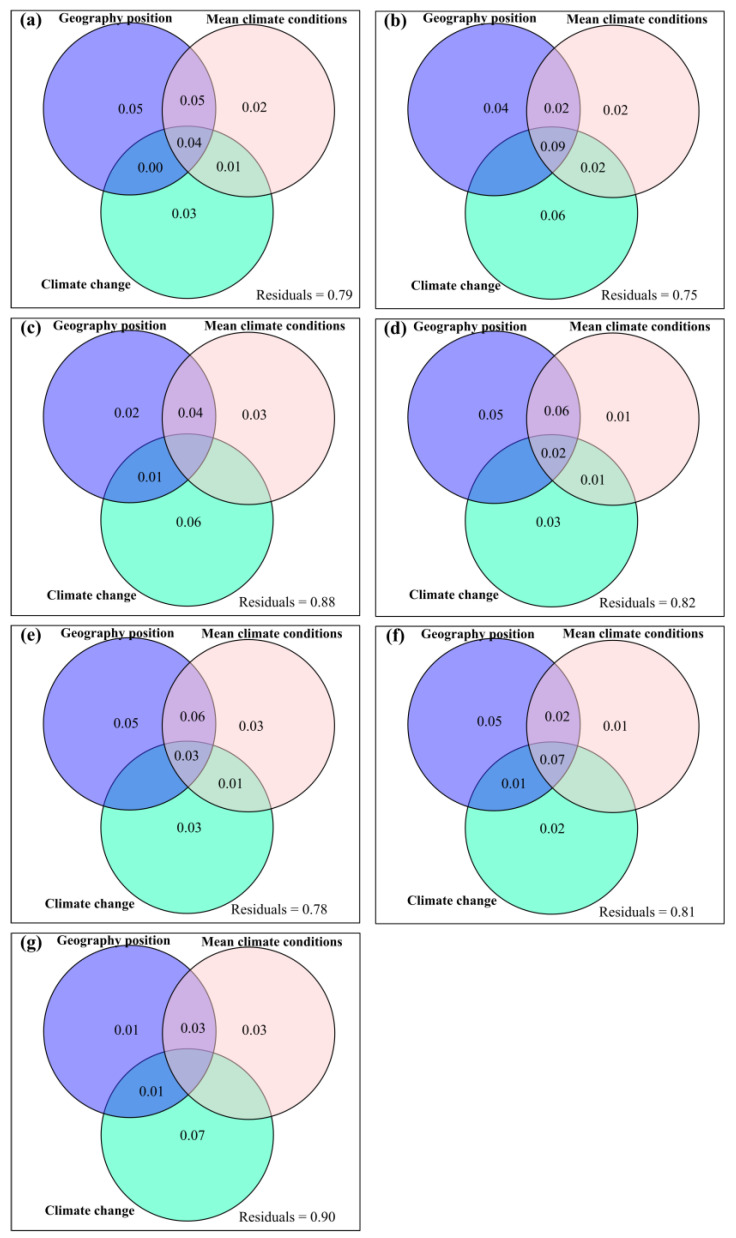
Relative influences of geography position, mean climate conditions and climate change on the variation rate for potential forage (**a**) nutrition storage, (**b**) crude protein storage, (**c**) crude ash storage, (**d**) ether extract storage, (**e**) acid detergent fiber storage, (**f**) neutral detergent fiber storage, and (**g**) water soluble carbohydrate storage.

**Figure 2:**
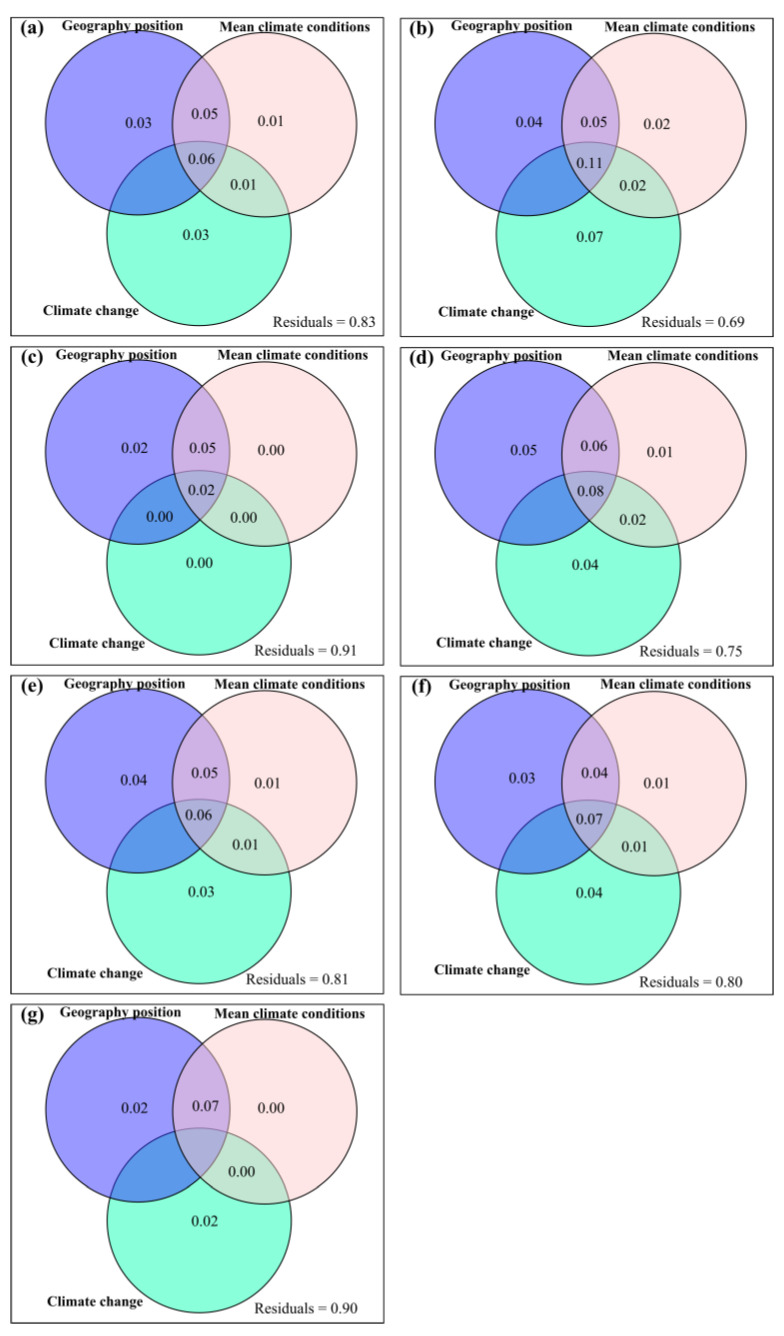
Relative influences of geography position, mean climate conditions and climate change on the variation rate for actual forage (**a**) nutrition storage, (**b**) crude protein storage, (**c**) crude ash storage, (**d**) ether extract storage, (**e**) acid detergent fiber storage, (**f**) neutral detergent fiber storage, or (**g**) water soluble carbohydrate storage.

**Figure A20:**
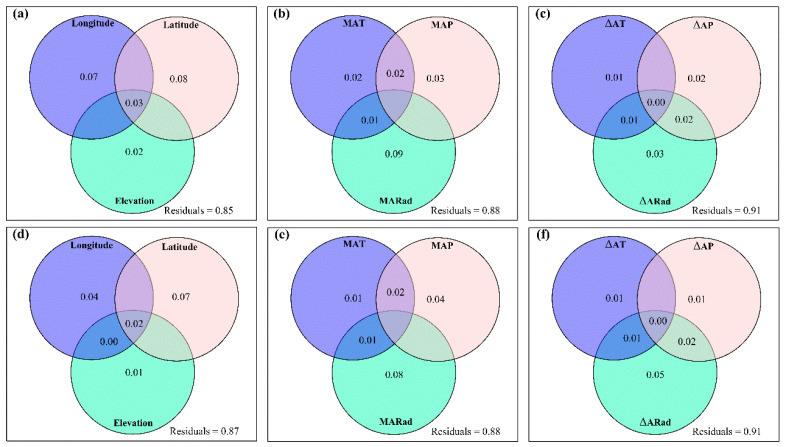
Relationships between (**a**–**c**) potential or (**d**–**f**) actual forage nutrition storage, and environmental variables.

Geographic position, mean climate conditions and climate change had some exclusive impacts on potential and actual forage nutrition storage, ΔCP_p_, ΔCP_a_, ΔAsh_p_, ΔAsh_a_, ΔEE_p_, ΔEE_a_, ΔADF_p_, ΔADF_a_, ΔNDF_p_, ΔNDF_a_, ΔWSC_p_ and ΔWSC_a_ (Figure 1 and Figure 2). Compared to longitude and elevation, latitude had greater exclusive impacts on potential and actual forage nutrition storage (Figure A20). Compared to MAT and MAP, MARad had greater exclusive impacts on potential and actual forage nutrition storage (Figure A20). Compared to ΔAT and ΔAP, ΔARad had the greater exclusive impacts on potential and actual forage nutrition storage (Figure A20).

### 2.2. Impacts of Anthropogenic Activities on Forage Nutrition Storage, and Their Correlations with Environmental Variables

**Figure 3:**
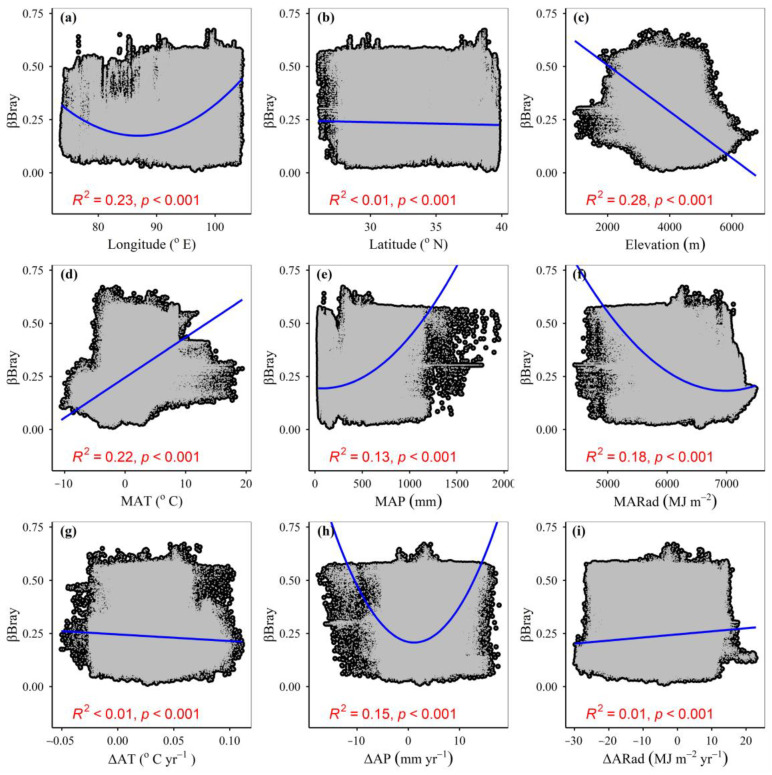
Relationships between the impacts of anthropogenic activities on forage nutrition storage (βBray), and (**a**) longitude, (**b**) latitude, (**c**) elevation, (**d**) mean annual temperature (MAT), (**e**) mean annual precipitation (MAP), (**f**) mean annual radiation (MARad), (**g**) change magnitude of annual temperature (ΔAT), (**h**) change magnitude of annual precipitation (ΔAP) and (**i**) change magnitude of annual radiation (ΔARad). The blue lines indicate the fitting lines.

**Figure 4:**
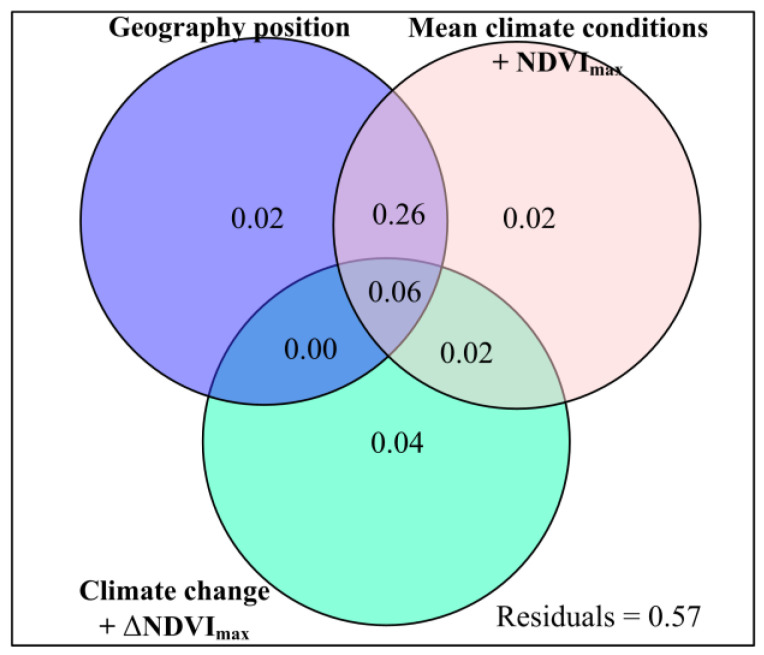
Relative impacts of geography position, mean climate conditions and maximum normalized difference vegetation index (mean climate conditions + NDVI_max_), and climate change and variation rate for maximum normalized difference vegetation index (climate change + ΔNDVI_max_) to the impacts of anthropogenic activities on forage nutritional storage (βBray).

**Figure A21:**
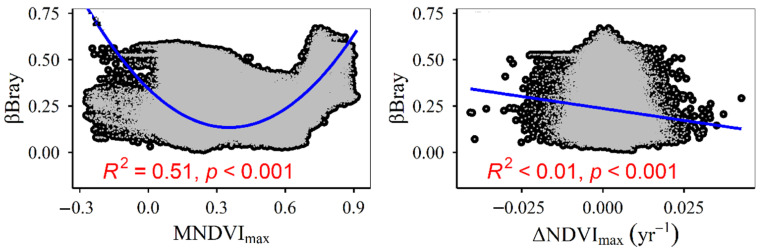
Relationships between the impacts of anthropogenic activities on forage nutrition storage (βBray), and maximum normalized difference vegetation index (NDVI_max_), linear change rate of NDVI_max_ (ΔNDVI_max_). The blue lines indicate the fitting lines.

**Figure A22:**
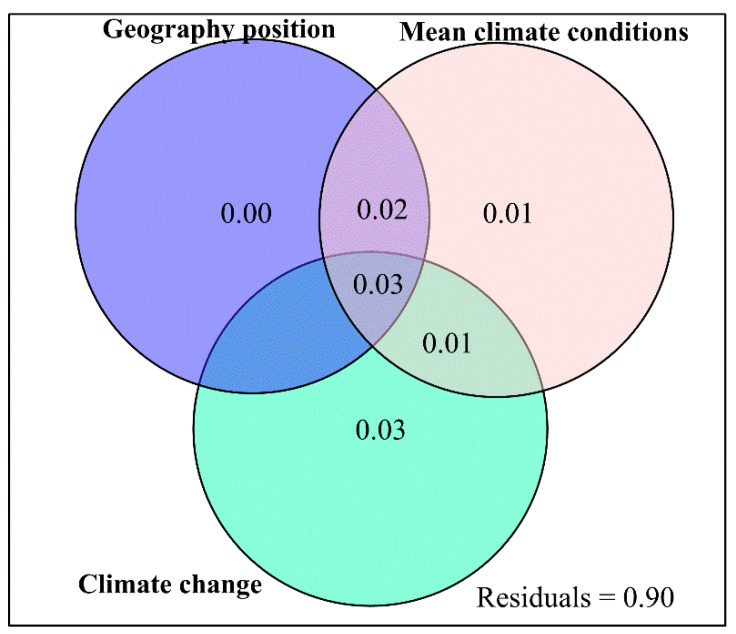
Relative contributions of geography position, mean climate conditions, and climate change to the impacts of anthropogenic activities on forage nutritional storage (left panel, βBray), and the linear change rate of βBray (right panel, ΔβBray).

**Figure A23:**
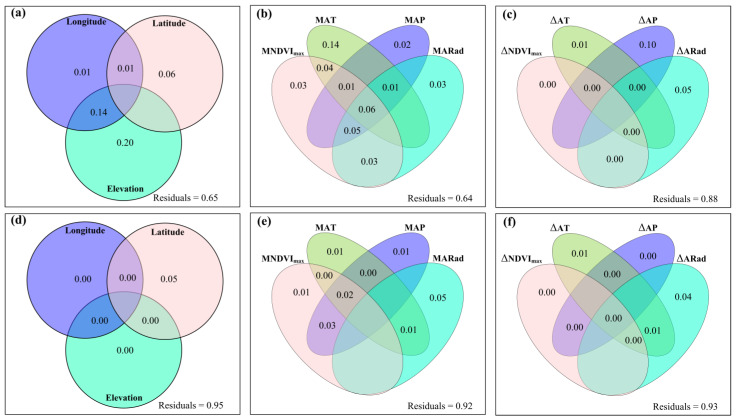
Relationships between (**a**–**c**) βBray or (**d**–**f**) ΔβBray, and environmental variables.

The βBray showed a significant (*p* < 0.001) quadratic correlation with longitude, MAP, MARad, MNDVI_max_ and ΔAP (Figure 3 and Figure A21). The βBray showed a significant (*p* < 0.001) positive correlation with MAT, but a significant (*p* < 0.001) negative correlation with elevation (Figure 3). Compared to geography position, and ‘mean climatic conditions’, ‘climate change’ had a greater exclusive influence on βBray (Figure A22). Compared to geography position, and ‘mean climatic conditions + NDVI_max_’, ‘climate change + ΔNDVI_max_’ had a greater exclusive influence on βBray (Figure 4). Compared to longitude and latitude, elevation had a greater exclusive impact on βBray (Figure A23a). Compared to MAP and MARad, MAT had a greater exclusive impact on βBray (Figure A23b). Compared to ΔAT and ΔARad, ΔAP had a greater exclusive impact on βBray (Figure A23c).

**Figure 5:**
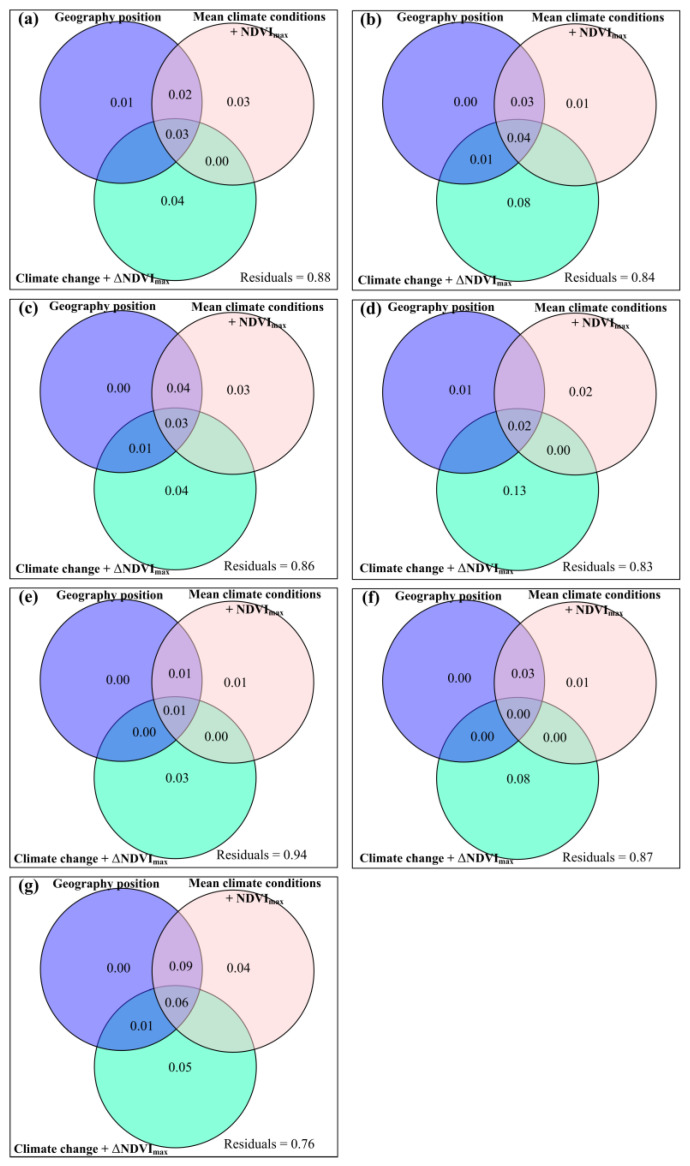
Correlations of the variation rate for the influence of anthropogenic activities on forage (**a**) nutrition storage, (**b**) crude protein storage, (**c**) crude ash storage, (**d**) ether extract storage, (**e**) acid detergent fiber storage, (**f**) neutral detergent fiber storage, and (**g**) water soluble carbohydrate storage, with geography position, mean climate conditions and maximum normalized difference vegetation index (mean climate conditions + NDVI_max_), and climate change and variation rate for maximum normalized difference vegetation index (climate change + ΔNDVI_max_).

**Figure A24:**
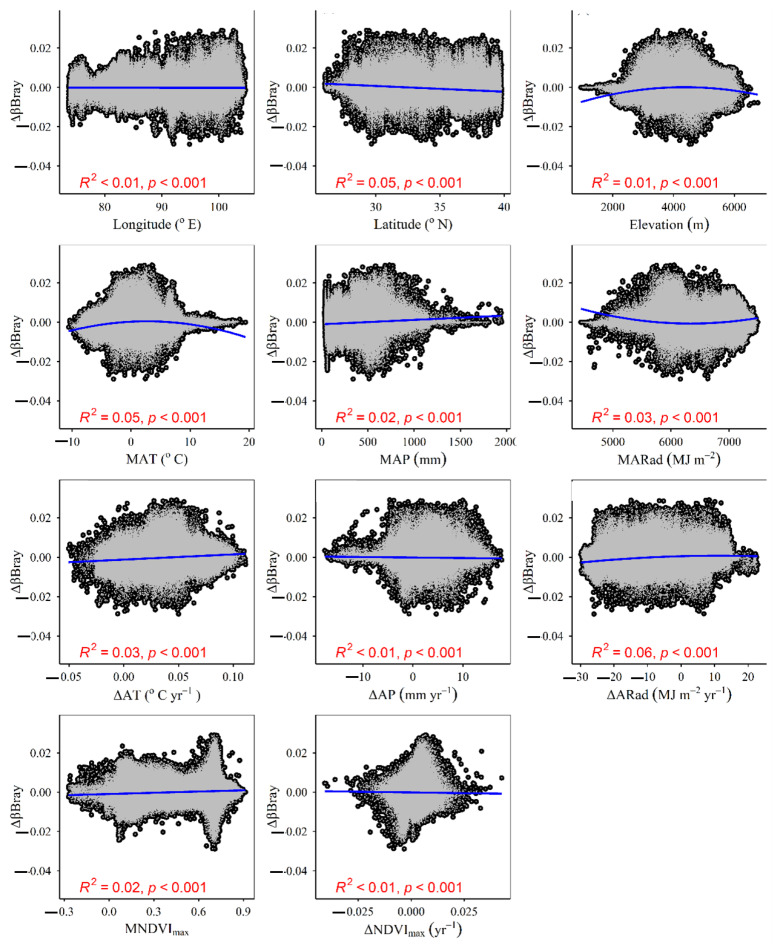
Correlations of the variation amplitudes for the response of forage nutrition storage to human activities (ΔβBray), and environmental variables. The blue lines indicate the fitting lines.

**Figure A25:**
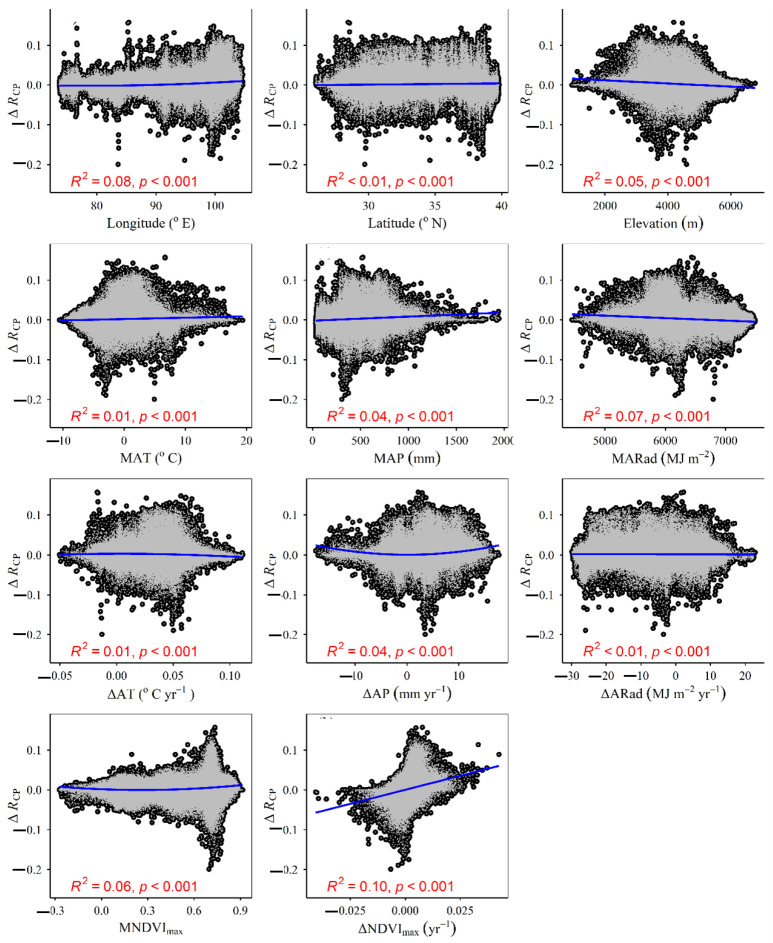
Correlations of the variation amplitudes for the response of forage crude protein to human activities, and environmental variables. The blue lines indicate the fitting lines.

**Figure A26:**
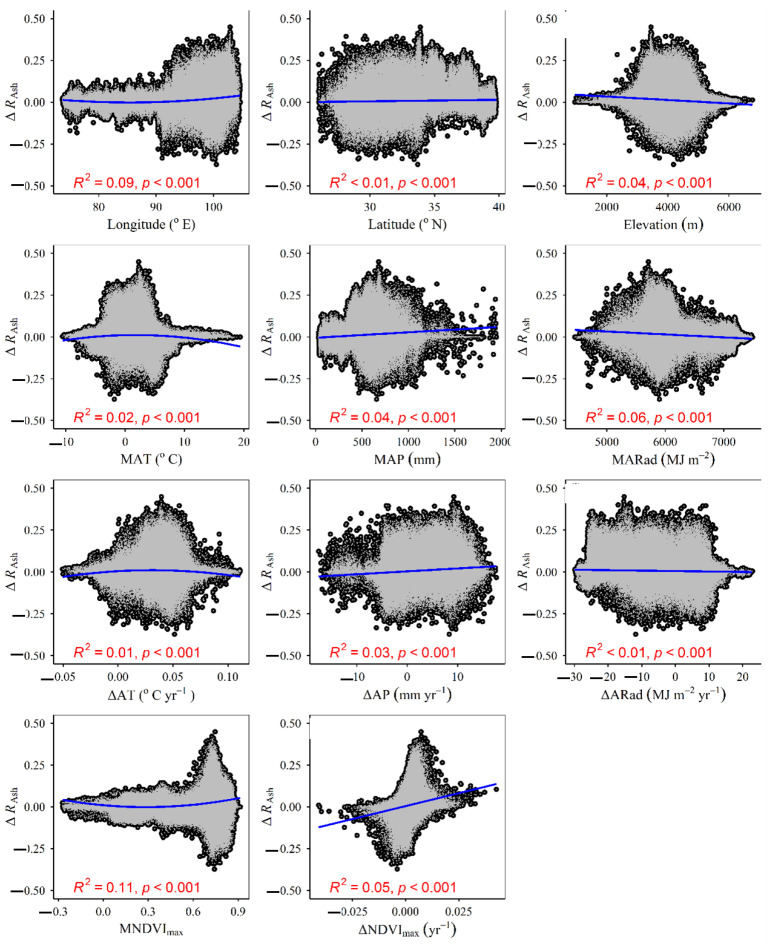
Correlations of the variation amplitudes for the response of forage crude ash to human activities, and environmental variables. The blue lines indicate the fitting lines.

**Figure A27:**
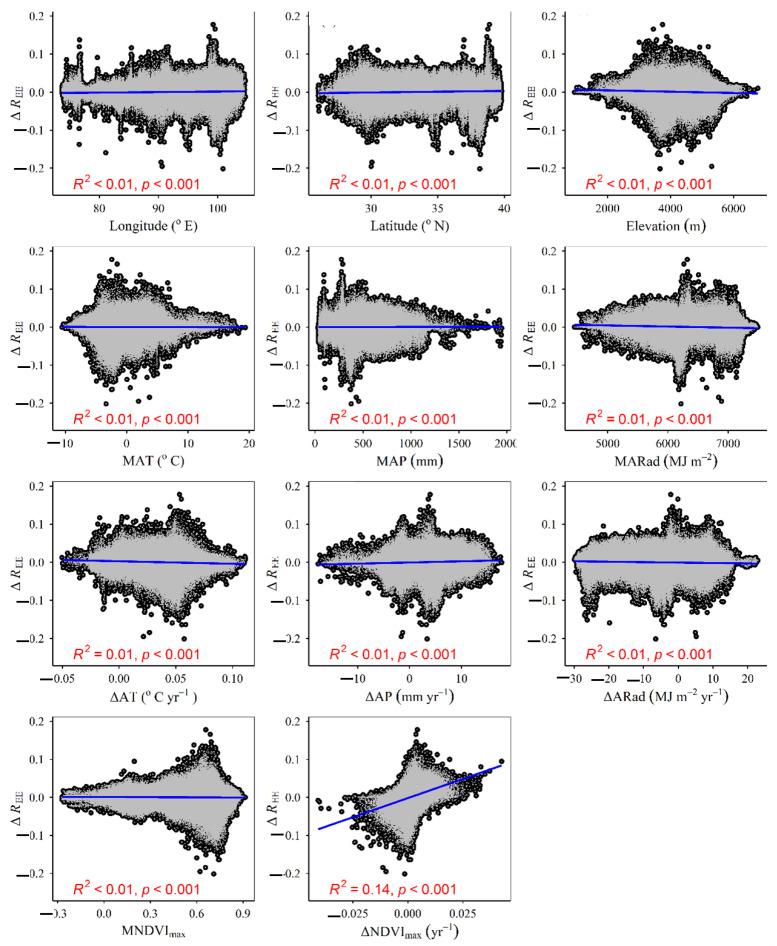
Correlations of the variation amplitudes for the response of forage ether extract to human activities, and environmental variables. The blue lines indicate the fitting lines.

**Figure A28:**
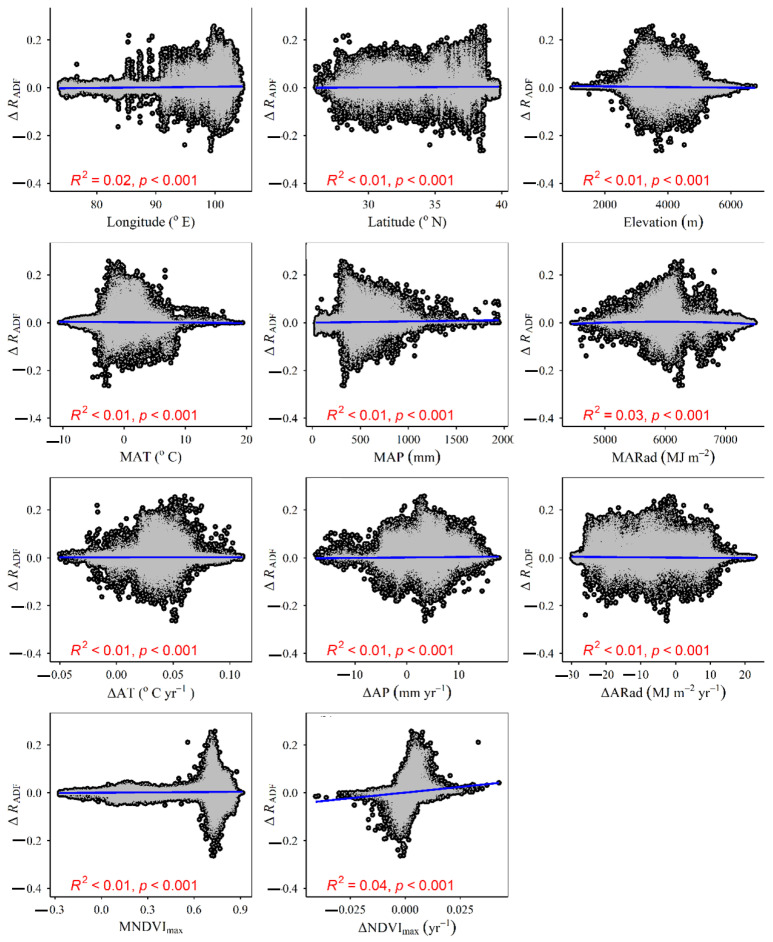
Correlations of the variation amplitudes for the response of forage acid detergent fiber to human activities, and environmental variables. The blue lines indicate the fitting lines.

**Figure A29:**
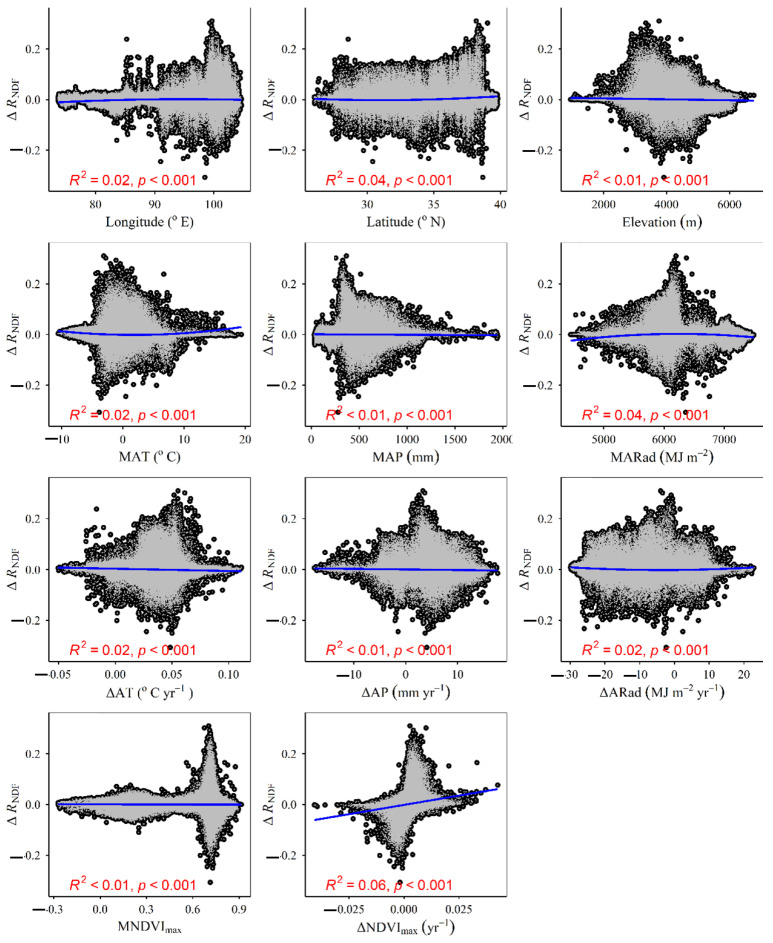
Correlations of the variation amplitudes for the response of forage neutral detergent fiber to human activities, and environmental variables. The blue lines indicate the fitting lines.

**Figure A30:**
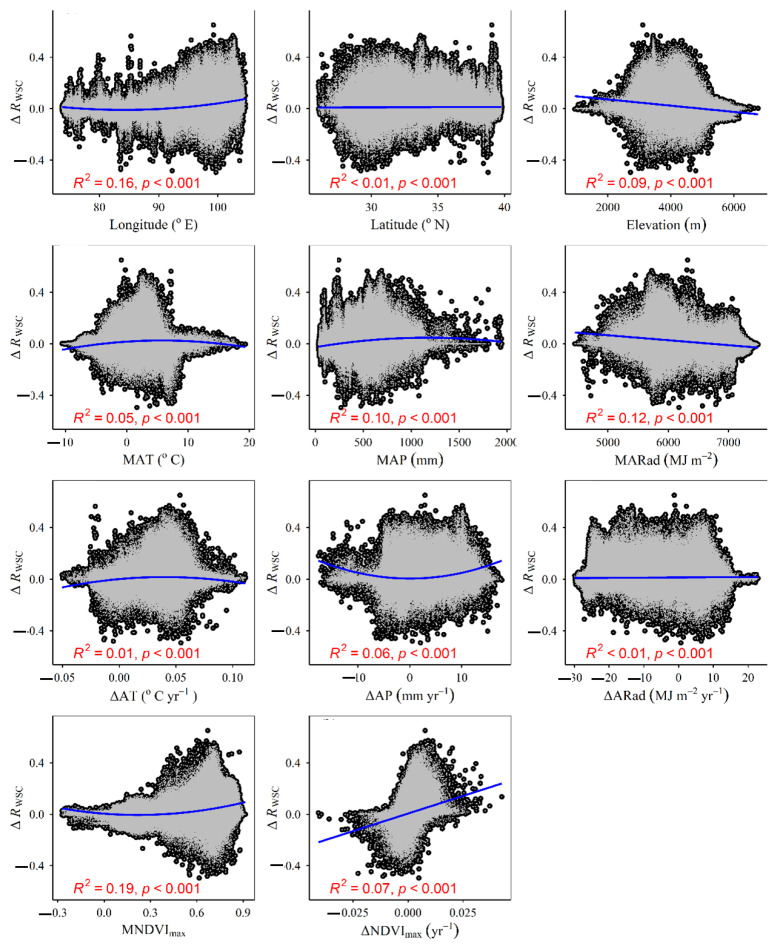
Correlations of the variation amplitudes for the response of forage water soluble carbohydrates to human activities, and environmental variables. The blue lines indicate the fitting lines.

The ΔβBray, Δ*R*_CP_, Δ*R*_Ash_, Δ*R*_EE_, Δ*R*_NDF_, Δ*R*_ADF_, and Δ*R*_WSC_ showed significant (*p* < 0.001) linear or non-linear relationships with longitude, latitude, elevation, MAT, MAP, MARad, MNDVI_max_, ΔAT, ΔAP, ΔARad and ΔNDVI_max_ (Figure A24, Figure A25, Figure A26, Figure A27, Figure A28, Figure A29 and Figure A30). Compared to geography position, and ‘mean climatic conditions’, ‘climate change’ had a greater exclusive influence on ΔβBray (Figure A22). Compared to geography position and ‘mean climatic conditions + NDVI_max_’, ‘climate change + ΔNDVI_max_’ had a greater exclusive influence on ΔβBray, Δ*R*_CP_, Δ*R*_Ash_, Δ*R*_EE_, Δ*R*_NDF_, Δ*R*_ADF_, and Δ*R*_WSC_ (Figure 5). Compared to longitude and elevation, latitude had a greater exclusive impact on ΔβBray (Figure A23d). Compared to MAP and MAT, MARad had a greater exclusive impact on ΔβBray (Figure A23e). Compared to ΔAT and ΔAP, ΔARad had a greater exclusive impact on ΔβBray (Figure A23f).

## 3. Discussion

### 3.1. Climate Change

Consistent with some earlier studies [15,24,25,26], some areas of the Tibetan Plateau did not become warmer but became cooler during the past two decades or so. That is, although the Tibetan Plateau is one of the most sensitive regions to climate change [27,28,29], both climate cooling and warming phenomena simultaneously occurred. The event was probably caused by a minimum in one of the later reasons. Firstly, solar radiation, as the primary energy source of surface temperature, can be one of the primary driver variables for surface temperature. All changes in surface albedo, cloud and precipitation can affect the solar radiation amount reaching the earth’s surface [30,31]. Surface albedo can be closely correlated to vegetation growth conditions [32,33]. Overall, vegetation productivity on the ‘Third Pole’ increased, while vegetation productivity decreased for some local areas [16]. This fact can result in an overall reduction in surface albedo, but a local increase in surface albedo [30]. The increase in surface albedo can increase the reflected solar radiation, which in turn may result in negative feedback on surface temperature. Increasing cloud amounts can reduce the amount of solar radiation reaching the surface and conversely can increase it. Precipitation can be often accompanied by an increase in cloud cover. Actually, solar radiation increased in some areas but decreased in other areas of the Tibetan Plateau [15]. Decreasing solar radiation (i.e., dimming) may cause a decline in surface temperature (i.e., cooling), and conversely can increase it (i.e., warming). Secondly, plant evapotranspiration may have a certain cooling influence on the earth’s surface, which in turn may result in negative feedback on surface temperature [30]. Vegetation growth conditions can affect vegetation evapotranspiration to some extent [30]. Vegetation growth conditions changed with altitude, latitude and elevation [34], which in turn can result in different changes in vegetation evapotranspiration among different areas under climate change on the ‘Third Pole’.

Some earlier research showed that climate cooling and warming can have asymmetrical influences on grassland ecosystems or alpine ecosystems [18,35,36,37]. For example, the first flower date can have asymmetrical responses to cooling and warming in an alpine meadow on the ‘Third Pole’ [17]. The forage nutrition quality of alpine grasslands can also have asymmetrical responses to cooling and warming in Tibet [15]. This event was strengthened by this study and is consistent with our hypothesis (*H1*). Consistent with some earlier studies [38,39], some areas of the Tibetan Plateau did not become wetter but even became dryer during the past two decades or so. This non-uniform precipitation change may result in the asymmetrical responses in forage nutrition storage of grasslands to increased precipitation and decreased precipitation on the Tibetan Plateau, which was similar to some earlier research [40,41] and consistent with our hypothesis (*H1*). This study found that radiation dimming and brightening can have asymmetrical influences on forage nutrition storage of grasslands on the Tibetan Plateau, which was similar to some earlier studies [29,42] and consistent with our hypothesis (*H1*).

As is well known, cold stress and dry stress can be two key limiting variables for plants on the Tibetan Plateau, and plants can be expected to be more active under climate warming or increased precipitation scenes, but be more inactive under cooling or decreased precipitation scenes [20,21,43,44,45]. Light saturation and photoinhibition phenomenon of plants can generally occur on the ‘Third Pole’ [21,46], and thus plants may be expected to be more active under certain dimming scenes [15,42]. However, the current study found that climate warming, increased precipitation, dimming did not always increase forage nutrition storage, cooling decreased precipitation, and brightening also did not always decrease forage nutrition storage. This event was inconsistent with our hypothesis (*H2*), and in line with earlier research [15] and might be caused by a minimum in one of the later phenomena. Firstly, warming may dampen low-temperature stress on plants, but aggravate drying stress on plants [44,47]. Occasionally, climate warming and drought may even simultaneously occur [15]. Climate warming may also aggravate the photoinhibition of plants [48] and reduce the temperature sensitivity of plants [23,49]. On the other hand, cooling might aggravate frigid stress on plants, but increase the temperature sensitivity of plants [23]. Secondly, dimming and increased precipitation often simultaneously occurred [31]. Increased precipitation may alleviate arid stress on plants, and dimming may dampen the photoinhibition of plants [24,42]. In contrast, increased precipitation or dimming may aggravate frigid stress in plants [31,47,50].

Some earlier studies found that the temperature sensitivity of plant productivity in grassland/alpine ecosystems generally declined with a warming magnitude [23,51], whereas other earlier studies found quite contrary results [24,52]. Moreover, recent research showed that a warming magnitude had non-linear influences on the temperature sensitivity of forage nutrition quality in Tibet [15]. In the current study, the response of forage nutrition storage appeared to be not always negatively correlated with a warming magnitude and may even increase with a warming magnitude for some cases, which was inconsistent with our hypothesis (*H3*). This event further cautioned that there was not a simple decreasing correlation between warming magnitude and the temperature sensitivity of plant productivity in grassland/alpine ecosystems and was probably caused by a minimum of the later phenomena. Firstly, as is well known, there can be three temperature basis points (i.e., minimum, maximum and optimal temperatures) for plant activities (e.g., plant turn greening) [44,53]. When the environmental temperature was within the ranges of minimum and optimal temperatures, the temperature sensitivity of plant activities probably increased with increasing warming magnitude. In contrast, when the environmental temperature was within the ranges of optimal and maximum temperatures, the temperature sensitivity of plant activities probably decreased with increasing warming magnitude. Secondly, there was probably an optimal, lowest and greatest warming magnitude for plant activities on the Tibetan Plateau [47]. When the warming magnitude was lower than the minimum warming magnitude or greater than the maximum warming magnitude, warming possibly did not increase plant productivity, but even reduced plant productivity. Thirdly, the temperature sensitives of plant productivity were also affected by plant productivity itself, water availability and elevation [23]. The warming influence on plant productivity was nonlinearly correlated with warming magnitude [47]. Generally, water availability declined with a warming magnitude [47]. Warming magnitude can also vary with elevation [54].

### 3.2. Anthropogenic Activities

The current study found that climate change and plant change had greater exclusive impacts on the variation in the response of forage nutrition storage to anthropogenic activities (i.e., ΔβBray). This event was consistent with our hypothesis (*H4*) and probably caused by a minimum of the latter phenomena. Firstly, climate change and plant change predominated the variation in the response of forage nutrition quality to anthropogenic activities in Tibet [15]. Secondly, anthropogenic activities increased the relative influence of climate change on forage nutrition storage but decreased the relative influences of mean climate conditions and geography position on forage nutrition storage (Figure 1 and Figure 2). Thirdly, azonal vegetation can be widely distributed. For example, there can be alpine meadows in the upper Brahmaputra River. Azonal vegetation can generally not coincide with a larger climatic zone and specific geography location, which in turn can dampen the influences of both mean climate conditions and geography position on forage nutrition storage.

The current study found that both mean climate conditions and geography positions can exclusively regulate the response of forage nutrition storage to anthropogenic activities. This event was consistent with our hypothesis (*H4*) and probably caused by a minimum of the latter phenomena. Firstly, both mean climate conditions and geography position can exclusively regulate the response of forage nutrition quality to anthropogenic activities in Tibet [15]. Secondly, zonal vegetation can fully reflect the vegetation type in a specific region and coincide with mean climate conditions and geography positions. The climatic conditions of the land surface can change with latitude, longitude and elevation, and the vegetation types can be zonally distributed in these three directions. For example, there can be alpine meadows, alpine steppes and desert steppes from east to west on the Tibetan Plateau [37,39,52]. Alpine steppe meadows and alpine meadows can be distributed from low elevation to high elevation in Northern Tibet [53]. Different vegetation types can probably have different nutrition quality and, in turn, forage storage. Thirdly, the environmental conditions where humans choose to live should be probably often related to climatic conditions and geographical location. There likely can be a limitation to the tolerance of humans and livestock to harsh climatic conditions, and thus humans can try to live in places with better climatic conditions and better vegetation growth conditions in order to gain advantages and avoid disadvantages. Generally, the higher the altitude and/or latitude are, the lower the temperature was, which in turn may reduce the intensity of anthropogenic activities [15,19]. Precipitation can decline from east to west in Tibet, which in turn may reduce the intensity of anthropogenic activities [15,55]. In the current study, the response of forage nutrition storage to anthropogenic activities indeed declined with decreasing temperature and increasing elevation (Figure 3).

## 4. Materials and Methods

### 4.1. Data

Earlier research introduced the data acquisition (e.g., NDVI_max_: growing season maximum normalized difference vegetation index; AT: annual temperature; AP: annual precipitation; ARad: annual radiation) in detail [8,15,56]. Kjeldahl, Soxhlet extraction, complete combustion, Van Soest and anthrone-based methods were used to analyze crude protein (CP), ether extract (EE), crude ash (Ash), neutral detergent fiber (NDF)/acid detergent fiber (ADF), and water-soluble carbohydrate (WSC) [8]. The data matrix of the forage CP pool, Ash pool, EE pool, NDF pool, ADF pool and WSC pool was the forage nutrition storage. The potential CP, Ash, EE, NDF, ADF and WSC pools were labeled by CP_p_, Ash_p_, EE_p_, NDF_p_, ADF_p_ and WSC_p_, respectively. The actual CP, Ash, EE, NDF, ADF and WSC pools were labeled by CP_a_, Ash_a_, EE_a_, NDF_a_, ADF_a_ and WSC_a_, respectively. The predicted accuracy of CP_p_, Ash_p_, EE_p_, NDF_p_, ADF_p_, WSC_p_, CP_a_, Ash_a_, EE_a_, NDF_a_, ADF_a_ and WSC_a_ with a relative bias of <6.00% and RMSE of <4.50 g m^−2^, were high [8]. The longitude, latitude, elevation, AT, AP, ARad, NDVI_max_, CP_p_, Ash_p_, EE_p_, NDF_p_, ADF_p_, WSC_p_, CP_a_, Ash_a_, EE_a_, NDF_a_, ADF_a_ and WSC_a_ had a spatial resolution of 1000 m × 1000 m and a temporal range of 2000–2020. The spatial range covered all grassland regions, including the Tibetan Plateau. Mean annual precipitation (MAP), mean annual temperature (MAT) and mean annual radiation (MARad) were referred to as the mean AP, AT and ARad in 2000–2020, respectively.

### 4.2. Statistical Analyses

The sens.slope function of trend package was used to calculate the change in AP (ΔAP), AT (ΔAT), ARad (ΔARad), NDVI_max_ (ΔNDVI_max_), CP_p_ (ΔCP_p_), Ash_p_ (ΔAsh_p_), EE_p_ (ΔEE_p_), NDF_p_ (ΔNDF_p_), ADF_p_ (ΔADF_p_), WSC_p_ (ΔWSC_p_), CP_a_ (ΔCP_a_), Ash_a_ (ΔAsh_a_), EE_a_ (ΔEE_a_), NDF_a_ (ΔNDF_a_), ADF_a_ (ΔADF_a_) and WSC_a_ (ΔWSC_a_) during 2000–2020. Univariate regression analysis was used to analyze the correlations of ΔCP_p_, ΔAsh_p_, ΔEE_p_, ΔNDF_p_, ΔADF_p_, ΔWSC_p_, ΔCP_a_, ΔAsh_a_, ΔEE_a_, ΔNDF_a_, ΔADF_a_ and ΔWSC_a_ with the three geographical position (i.e., latitude, longitude, elevation), three mean climate conditions (i.e., MAP, MAT and MARad) and three climate change variables (i.e., ΔAP, ΔAT, ΔARad), respectively. The varpart function of the vegan package was used to partition the variation of ΔCP_p_, ΔAsh_p_, ΔEE_p_, ΔNDF_p_, ΔADF_p_, ΔWSC_p_, ΔCP_a_, ΔAsh_a_, ΔEE_a_, ΔNDF_a_, ΔADF_a_ and ΔWSC_a_ into mean climate conditions, geography positions and climate change. The βBray value (i.e., βBray) between the data matrix of CPp, Ashp, EEp, NDFp, ADFp and WSCp, and the data matrix of CPa, Asha, EEa, NDFa, ADFa and WSCa was treated as the influence of anthropogenic activities on forage nutrition storage. Univariate regression analysis was used to analyze the correlation of the βBray with elevation, latitude, longitude, MAP, MAT and MARad, respectively. The varpart function was used to partition the βBray into geography positions, mean climate conditions + NDVI_max_, and climate change + ΔNDVI_max_. The ratio of CP_a_ versus CP_p_ (*R*_CP_), Ash_a_ versus Ash_p_ (*R*_Ash_), EE_a_ versus EE_p_ (*R*_EE_), NDF_a_ versus NDF_p_ (*R*_NDF_), ADF_a_ versus ADF_p_ (*R*_ADF_), and WSC_a_ versus WSC_p_ (*R*_WSC_) was calculated, respectively. The sens.slope function of the trend package was also used to calculate the change in the βBray (ΔβBray), *R*_CP_ (Δ*R*_CP_), *R*_Ash_ (Δ*R*_Ash_), *R*_EE_ (Δ*R*_EE_), *R*_NDF_ (Δ*R*_NDF_), *R*_ADF_ (Δ*R*_ADF_), and *R*_WSC_ (Δ*R*_WSC_), respectively. Univariate regression analysis was used to analyze the correlation of Δ*R*_CP_, Δ*R*_Ash_, Δ*R*_EE_, Δ*R*_NDF_, Δ*R*_ADF_ and Δ*R*_WSC_ with elevation, latitude, longitude, MAP, MAT, MARad, ΔAP, ΔAT and ΔARad, respectively. The varpart function was used to partition the ΔβBray, Δ*R*_CP_, Δ*R*_Ash_, Δ*R*_EE_, Δ*R*_NDF_, Δ*R*_ADF_ and Δ*R*_WSC_ into geography position, mean climate conditions + NDVI_max_, and climate change + ΔNDVI_max_, respectively.

## 5. Conclusions

Here, the impacts of anthropogenic activities and climate change on forage nutrition storage were investigated in the grasslands of the Tibetan Plateau from 2000 to 2020. Three cautions emerged from the current study. Firstly, cooling and drying did not always reduce forage nutrition storage, and warming and wetting also did not always increase forage nutrition storage. Secondly, on one hand, compared to the local temperature and precipitation, local radiation had greater contributions to the change in potential and actual forage nutrition storage. On the other hand, compared to precipitation and temperature changes, radiation change had greater contributions to the change in potential and actual forage nutrition storage. Thirdly, on the one hand, compared to local temperature and precipitation, local radiation had greater contributions to the change in the impacts of humankind activities on forage nutrition storage (i.e., ΔβBray). On the other hand, compared to precipitation and temperature change, radiation change had greater contributions to the change in ΔβBray. These warnings can have important theoretical and practical values for the nutrient balance of livestock, the balance of forage livestock, and the optimal management of grassland ecosystems, at least for the Tibetan Plateau.

## Data Availability

The datasets generated for this study are available on request from the corresponding author.
